# Chk1-mediated phosphorylation of Cdh1 promotes the SCF^βTRCP^-dependent degradation of Cdh1 during S-phase and efficient cell-cycle progression

**DOI:** 10.1038/s41419-020-2493-1

**Published:** 2020-04-28

**Authors:** Debjani Pal, Adrian E. Torres, Benjamin R. Stromberg, Abbey L. Messina, Andrew S. Dickson, Kuntal De, Belinda Willard, Monica Venere, Matthew K. Summers

**Affiliations:** 1 Department of Radiation Oncology, Arthur G James Comprehensive Cancer Center and Richard L. Solove Research Institute, The Ohio State University Medical Center, Columbus, OH 43210 USA; 2 Department of Cancer Biology, Lerner Research Institute, Cleveland Clinic, Cleveland, OH 44195 USA; 3 Proteomics and Metabolomics Core, Lerner Research Institute, Cleveland Clinic, Cleveland, OH 44195 USA; 4 Present Address: Bioscience Division, Oak Ridge National Lab, Oak Ridge, TN 37830 USA

**Keywords:** Checkpoints, DNA synthesis, Phosphorylation, Ubiquitylation, Ubiquitin ligases

## Abstract

APC/C^Cdh1^ is a ubiquitin ligase with roles in numerous diverse processes, including control of cellular proliferation and multiple aspects of the DNA damage response. Precise regulation of APC/C^Cdh1^ activity is central to efficient cell-cycle progression and cellular homeostasis. Here, we have identified Cdh1 as a direct substrate of the replication stress checkpoint effector kinase Chk1 and demonstrate that Chk1-mediated phosphorylation of Cdh1 contributes to its recognition by the SCF^βTRCP^ ubiquitin ligase, promotes efficient S-phase entry, and is important for cellular proliferation during otherwise unperturbed cell cycles. We also find that prolonged Chk1 activity in late S/G2 inhibits Cdh1 accumulation. In addition to promoting control of APC/C^Cdh1^ activity by facilitating Cdh1 destruction, we find that Chk1 also antagonizes activity of the ligase by perturbing the interaction between Cdh1 and the APC/C. Overall, these data suggest that the rise and fall of Chk1 activity contributes to the regulation of APC/C^Cdh1^ activity that enhances the replication process.

## Introduction

Proper progression of the cell cycle is driven by the timely degradation of cell-cycle regulators mediated by the ubiquitin proteasome system (UPS), which is necessary to maintain the systematic and coordinated duplication and subsequent segregation of the genome that is required to maintain its integrity^1–6^. Cells also possess a number of cell-cycle checkpoints that work in conjunction with the UPS to further ensure orderly cell-cycle progression and genome stability. Any defects in these processes may lead to irreversible damage including genetic alteration, developmental defects, and cancer.

There are over 600 different ubiquitin ligase in the human genome. Among them, the Skp1-Cullin-F-Box (SCF) complexes and the Anaphase Promoting Complex/Cyclosome (APC/C) are best known for their roles in cell-cycle control^7^. For SCF complexes, the F-box protein determines substrate specificity and substrate recognition and is often dependent on post-translational modification of the substrate. For the majority of the well-studied F-boxes (e.g., Skp2, Fbw7, βTRCP) it is substrate phosphorylation that allows efficient recognition by the F-box protein^2,8^. Similar to the SCF, APC/C activity and substrate recognition depends on either one of two WD40 repeat proteins, Cdc20 or Cdh1^9–11^. While APC/C^Cdc20^ participates almost exclusively in mitotic progression, the biological function of APC/C^Cdh1^ is much more complex. The primary functions of APC/C^Cdh1^ include mitotic exit, G1 maintenance, quiescence, and differentiation^6,12,13^. Cdh1 plays key roles in the maintenance of chromosomal integrity and genomic stability^14–21^. In keeping with its diverse functions, Cdh1 has been established as a tumor suppressor as Cdh1-deficient mice exhibit genomic instability and develop epithelial tumors^16^. Indeed, many known Cdh1 substrates are overexpressed in various cancers with high genomic instability and are associated with oncogenesis^12,16,22,23^. Thus, dysregulation of APC/C^Cdh1^ could play a significant role in carcinogenesis.

Multiple mechanisms work together to keep the activity of APC/C^Cdh1^ low from late G1 until late mitosis to allow for the accumulation of cell-cycle drivers that are regulated by APC/C^Cdh1^^5^. Notably, as APC/C^Cdh1^ activity diminishes, the accumulation of substrates such as Cdc25A, Skp2, and Cyclin A promote phosphorylation of Cdh1 by Cyclin-Cdk complexes, which further weakens APC/C^Cdh1^ activity by disrupting the interaction of Cdh1 with APC/C^9,24–30^. Degradation of Cdh1 in late G1 is mediated by APC/C^Cdh1^ and SCF^Cyclin F^^31,32^. In addition, sequential phosphorylation of Cdh1 by both Cyclin A-Cdk2 and Plk1 leads to its degradation via SCF^βTRCP^^33^. Together these mechanisms cooperate to maintain low APC/C^Cdh1^ activity during S,G2, and early mitosis to ensure efficient cell-cycle oscillation.

One remaining question regarding the degradation of Cdh1 in late G1/S is why Cdh1 is targeted by SCF^βTRCP^ at this stage of the cell cycle when the activities of Cyclin A-Cdk, and particularly Plk1, are at their lowest, but is not targeted in G2 when the activities of these kinases are maximal. We therefore speculated that an additional, S-phase active kinase would be involved. The Chk1 kinase was recently implicated in the regulation of Cdh1 following replication stress and several lines of evidence indicated Chk1 as a candidate Cdh1 kinase^34^. Chk1 is required for the cellular response to replication stress including promoting the SCF^βTRCP^-mediated degradation of Cdc25A^35,36^. Chk1 is also central to the normal control of the replication program during S-phase and early activation of Chk1 is associated with premature S-phase entry, similar to loss of APC/C^Cdh1^ activity^37–39^. Finally, APC/C^Cdh1^ negatively regulates Chk1 activitation, suggesting that a feedback loop between these proteins may exist^15,37,40,41^. Herein, we determined that Chk1 phosphorylates Cdh1 promoting its recognition by SCF^βTRCP^, ubiquitination, and subsequent proteasome-mediated degradation. We further defined that expressing a constitutively active Chk1 attenuates Cdh1 accumulation in G2 phase. Additionally, we find that phosphorylation by Chk1 perturbs APC/C^Cdh1^ complex formation. Together our data provide a model whereby Chk1 activity in S-phase cooperates with Cyclin A-Cdk2 and low Plk1 activity to limit APC/C^Cdh1^ activity whereas loss of Chk1 activity in G2 permits Cdh1 accumulation and APC/C^Cdh1^ complex formation despite increasing Plk1 and Cdk activity.

## Results

### Chk1 modulates Cdh1 stability

**Fig. 1:**
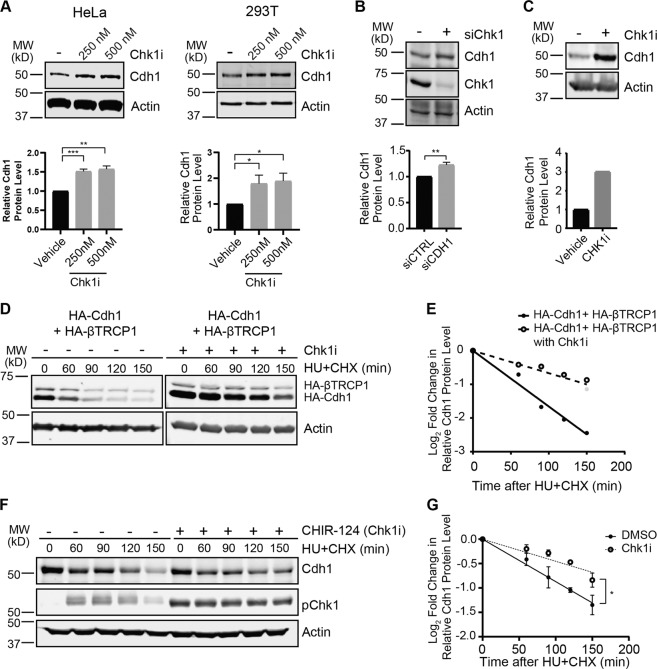
Chk1 modulates Cdh1 stability. **a** Inhibition of Chk1 increases the stability of endogenous Cdh1. Immunoblot analysis of whole-cell lysates derived from both HeLa and 293T cells treated with increasing doses of the Chk1 inhibitor (Chk1i), CHIR-124, for 5 h before harvesting. Lower panels, Cdh1 band intensities were normalized to actin and then further normalized to vehicle (*n* = 3). **b** Depletion of endogenous Chk1 leads to increased levels of endogenous Cdh1. Immunoblot analysis of whole-cell lysates derived from 293T cells transfected with the indicated siRNA (non-targeting siCTRL, −; siChk1, +). Lower panels, Cdh1 band intensities were normalized to actin and then further normalized to siCTRL transfected cells (*n* = 3). **c** Mitotic population does not contribute to the increased endogenous Cdh1 level after Chk1 inhibitor treatment. Immunoblot analysis of whole-cell lysates derived from HeLa cells after removal of the mitotic population via a mitotic shake-off post Chk1 inhibitor, CHIR-124 (500 nM) treatment for 5 h. Lower panel, Cdh1 band intensities were normalized to actin and then further normalized to vehicle-treated cells. **d**, **e** Inhibition of Chk1 increases the half-life of transfected Cdh1 in 293T cells. **d** Immunoblot analysis of whole-cell lysates derived from 293T cells, transfected with HA-Cdh1 and HA-βTRCP1 constructs. Cells were treated with both hydroxyurea (HU) and Chk1 inhibitor (Chk1i), CHIR-124 (500 nM), for another 4 h before addition of 50 µg/ml cycloheximide (CHX). At the indicated time points, whole-cell lysates were prepared for immunoblot analysis. **e** Quantification of the band intensities in (**d**). Cdh1 band intensities were normalized to actin and then further normalized to *t* = 0 controls. **f**, **g** Inhibition of Chk1 increases the half-life of endogenous Cdh1 in 293T cells. **f**. 293T cells were treated with both hydroxyurea (HU) and Chk1 inhibitor (Chk1i), CHIR-124 (500 nM), for another 4 h before addition of 50 µg/ml cycloheximide (CHX). At the indicated time points, whole-cell lysates were prepared for immunoblot analysis. **g** Quantification of the band intensities in (**f**) (*n* = 3). Mean and SEM are provided. Significance in panels (**a**), (**b**), and (**g**) was determined by a one-tailed unpaired *T*-test (*p < 0.05, **p < 0.005, and ***p < 0.0005).

Chk1 signaling promotes degradation of Cdh1^34^. However, whether Chk1 acts directly upon Cdh1 to elicit degradation has not been demonstrated. We examined the impact of Chk1 on Cdh1 levels. Asynchronously dividing HeLa and 293T cells showed increased endogenous Cdh1 protein upon inhibition of Chk1 with CHIR-124 (Chk1i) (Fig. 1a)^42^. Similarly, depletion of Chk1 in asynchronous 293T cells increased endogenous Cdh1 levels (Fig. 1b). Furthermore, as Chk1 inhibition could allow cells to progress to mitosis, where Cdh1 stability is increased, we determined that Chk1 inhibition did not alter the cell cycle (Fig. S1a) and that Cdh1 protein levels are elevated in interphase cells after Chk1 inhibition by mechanically removing mitotic cells (Fig. 1c). To evaluate the relationship between Chk1 and Cdh1 stability, we induced replication stress, which is known to reduce the half-life of Cdh1, with hydroxyurea (HU)^34,43^. Inhibition of Chk1 led to an increase in the stability of both exogenously expressed Cdh1 (Fig. 1d, e) and endogenous Cdh1 (Fig. 1f, g) even in the presence of hydroxyurea-induced replication stress. These data show that Chk1 is involved in the regulation of Cdh1 protein abundance under conditions of replication stress and in unperturbed proliferating cells.

### SCF^βTRCP1^ negatively regulates Cdh1 at the G1/S boundary in a Chk1-dependent manner

**Fig. 2:**
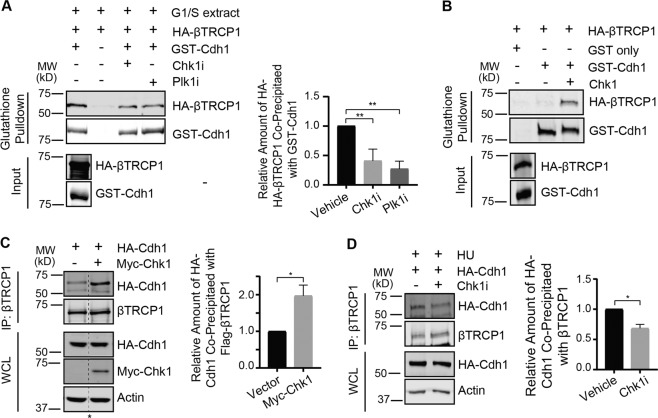
SCF^βTRCP1^ negatively regulates Cdh1 at the G1-S boundary in a Chk1-dependent manner. **a**, **b** Chk1 impacts on interaction between βTRCP1 and Cdh1 both in G1-S extracts and in vitro. **a** HeLa cells were treated with HU (2 mM) for 20 h to arrest them at the G1-S boundary and extracts were prepared. The G1-S extracts were treated with Chk1 inhibitor, CHIR-124 (500 nM) where indicated before incubating with GST-Cdh1. After phosphorylation, GST-Cdh1 was first bound by glutathione beads and then mixed with in vitro translated HA-βTRCP1 for an hour. Immunoblot analysis was carried out to detect the bound HA-βTRCP1 protein. Right panel, quantification of the band intensities. Co-precipiated HA-βTRCP1 band intensities were normalized to the respective GST-Cdh1 bands and then further normalized to vehicle control (-). Data are represented as mean ± SD, **p < 0.005 by ANOVA with Holm-Sidak post-test, *n* = 3. **b** Immunoblots showing that the in vitro phosphorylation of GST-Cdh1 by Chk1 promotes its binding with HA-βTRCP1. **c**, **d** Chk1 activity is crucial for the interaction of Cdh1 with βTRCP1 in vivo. **c** Immunoblot analysis of immunoprecipitates and whole-cell lysates derived from 293T cells transfected with HA-Cdh1 and Myc-Chk1 (where indicated). 30 h post-transfection, cells were treated with proteasome inhibitor MG132 (10 µM) for 5 h and then harvested to do immunoprecipitation to detect the interaction between HA-Cdh1 and endogenous βTRCP1 proteins. Right panel, quantification of the band intensities (*n* = 3). Immunoprecipiated HA-Cdh1 band intensities were normalized to the respective βTRCP1 IP bands and then further normalized to Vector control (-). The dashed line and asterisk represents the removal of an intervening lane btween these two samples. **d** Immunoblot analysis of immunoprecipitates and whole-cell lysates derived from 293T cells transfected with both HA-Cdh1. 30 h post-transfection, Cells were treated with hydroxyurea (HU), Chk1 inhibitor (where indicated), and the proteasome inhibitor MG132 (10 µM) for 5 h before harvesting to do IPs. Right panel, quantification of the band intensities (*n* = 3). Immunoprecipiated HA-Cdh1 band intensities were normalized to the respective βTRCP1 IP bands and then further normalized to Vehicle-treated control (-). Data are presented as mean and SEM. Significance in panel (**a**) was determined with a one-way ANOVA and significance in panels (**c**) and (**d**) was determined by a one-tailed unpaired *T*-test (*p < 0.05 and **p < 0.005).

Cdh1 is targeted by SCF^βTRCP^ in a Cyclin A-Cdk2 and Plk1-dependent manner to promote the G1/S transition^33^. However, as both kinases are not highly active during the G1/S transition, when Cdh1 stability is low, but are highly active in G2, when Cdh1 stability is increased, we speculated that an additional S-phase-active kinase might cooperate with these kinases to destabilize Cdh1. Chk1 plays a key role in the S-phase checkpoint and the replication process, suggesting Chk1 as a candidate kinase contribuiting to Cdh1 recognition by SCF^βTRCP^^44–52^. Given that replication stress promotes APC/C^Cdh1^ inactivation in a Chk1-dependent manner, we examined the role of Chk1 on the association of Cdh1 with βTRCP1. Inhibition of Chk1 in HeLa G1/S extracts with CHIR-124 reduced binding between GST-Cdh1 and HA-βTRCP1, similar to inhibition of Plk1 (Fig. 2a). We next showed that phosphorylation of Cdh1 by purified Chk1 (as indicated by reduced electrophoretic mobility of GST-Cdh1) induced binding to βTRCP1 in vitro (Fig. 2b). These data support that Chk1 promotes βTRCP1-induced degradation of Cdh1. We next determined the impact of Chk1 activity on the interaction of HA-Cdh1 and Flag-βTRCP1 in 293T cells. Co-expression of Myc-Chk1 increased the interaction between Cdh1 and βTRCP1 (Fig. 2c) whereas inhibition of endogenous Chk1 reduced the association (Fig. 2d). Similalry, the interaction between transfected Cdh1 and endogenous βTRCP1 is reduced by the use of a second Chk1 inhibitor, AZD7762 (Fig. S1b). Our biochemical data indicate a central role for Chk1-mediated phosphorylation of Cdh1 in its recognition by βTRCP1 and subsequent degradation.

### Phosphorylation of Cdh1 by Chk1 promotes recognition of Cdh1 by SCF^βTRCP1^

**Fig. 3:**
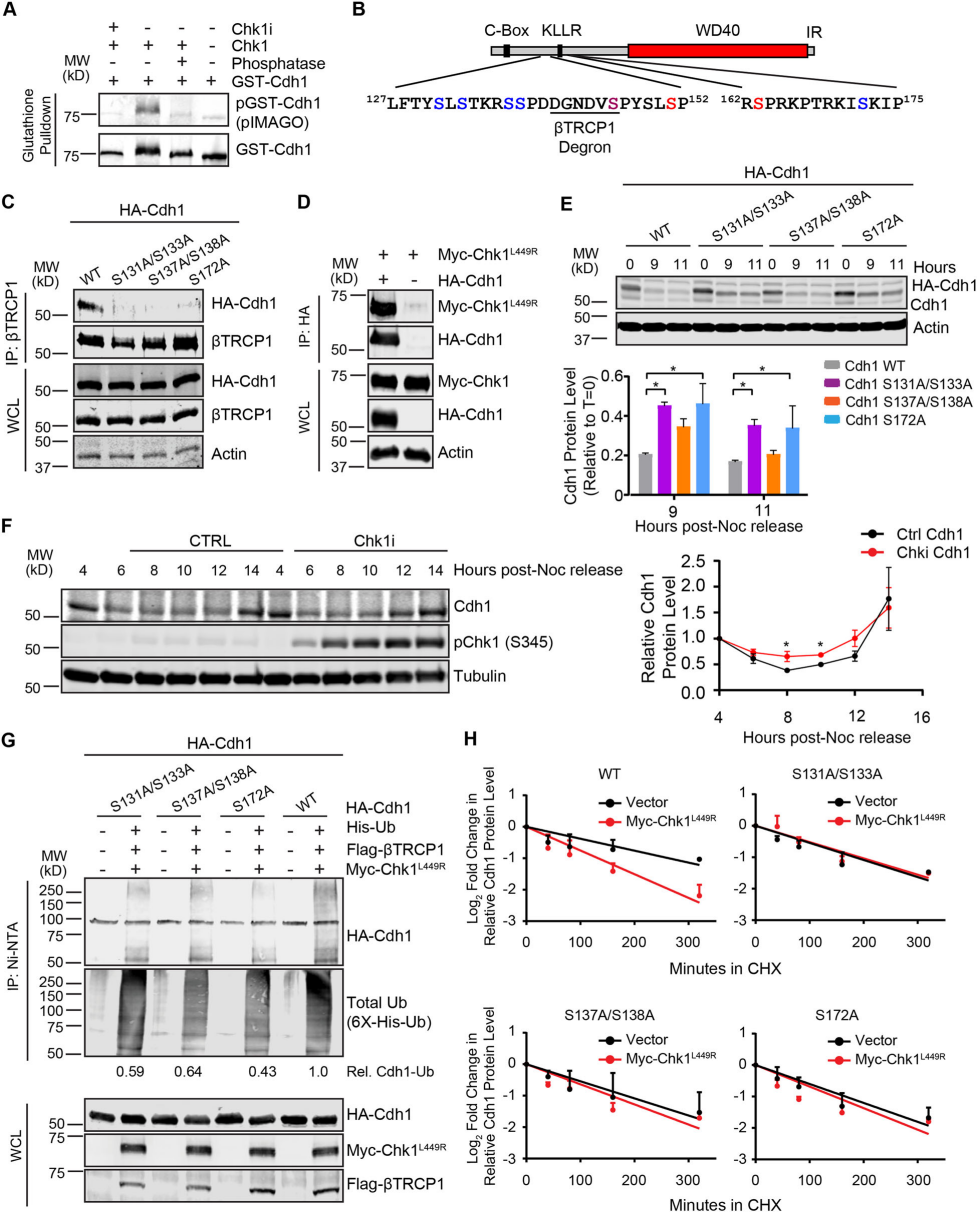
Phosphorylation of Cdh1 by Chk1 creates a phosphodegron recognized by SCF^βTRCP1^. **a** Chk1 phosphorylates Cdh1 in vitro. GST-Cdh1 was phosphorylated with Chk1 through in vitro kinase assays. Immunoblot represents immunoprecipitated GST-Cdh1 on glutathione beads following in vitro kinase assays. PIMAGO western blot kit was used to detect phospho-GST-Cdh1. Phosphorylation was further confirmed by including Chk1 inhibitor (Chk1i) where indicated during the kinase assays or by treating phosphorylated GST-Cdh1 with phosphatase. **b** Schematic diagram of Cdh1. Chk1-mediated phosphorylation sites identified from mass-spectrometry analysis are indicated in blue. Cdh1 phosphorylation sites mediated by Cyclin A-Cdk2 and Plk1 are in red and magenta, respectively. The SCF-βTRCP1 phosphodegron is indicated. **c** Chk1-mediated phosphorylation of Cdh1 creates a binding site for βTRCP1. 293T cells were transfected with the indicated HA-Cdh1 constructs. Cells were treated with the proteasome inhibitor MG132 (10 µM) for 5 h and the interaction between HA-Cdh1 and endogenous βTRCP1 was analyzed. **d** Cdh1 interacts with Chk1 in vivo. Both HA-Cdh1 and constitutively active Myc-Chk1^L449R^ were co-expressed in 293T cells. 30 h post-transfection, cells were treated with the proteasome inhibitor MG132 (10 µM) for 5 h. The interaction between HA-Cdh1 and Myc-Chk1^L449R^ proteins was monitored by immunoprecipitation. **e** Chk1-mediated phosphorylation regulates Cdh1 protein levels at the G1/S transistion. HeLa cells were transfected with the indicated doxycycline-inducible HA-Cdh1 constructs and synchronized at G1/S by double thymidine block (DTB). Doxycycline was added during the final 4 h of the DTB. Cells were released into nocodazole and mitotic cells were collected and relased into fresh media. Whole-cell lysates were prepared for immunoblot analysis at the indicated time points. The intensities of Cdh1 bands were normalized to actin, then normalized to the the level of the Cdh1 protein at *t* = 0 h. The plot represents the relative fraction of indicated Cdh1 remaining at the indicated time points. Data are represented as mean ± SEM, *p < 0.05 by ANOVA with Holm-Sidak post-test, *n* = 3. **f** Inhibition of endogenous Chk1 by Chk1 inhibitor, CHIR-124 (500 nM) affects Cdh1 level at the G1/S boundary. Hela cells were synchronized in mitosis with a thymidine-nocodazole block. 4 h after release from nocodazole into the cell cycle, cells were treated with Chk1 inhibitor, CHIR-124 (500 nM) where indicated and collected at specific time point for immunoblot analysis. The plot represents relative Cdh1 protein level where the Cdh1 bands were normalized to tubulin, then normalized to the *t* = 4 h time point. Data are represented as mean ± SD, *p < 0.05 by one-tailed unpaired *t*-test, *n* = 3. **g**, **h** Chk1 promotes ubiquitinatin by SCF^βTRCP1^ and destabilizes Cdh1. **g** In vivo ubiquitination assays shows that SCF^βTRCP1^ promotes Cdh1 ubiquitination in a Chk1-dependent manner. 293T cells were transfected with the constructs encoding HA-Cdh1 or HA-Cdh1 mutants, His-ubiquitin, Flag- βTRCP1 and Myc-Chk1^L449R^ as indicated. After a treatment with MG132 for 5 h, the lysates were collected and incubate with Ni-NTA agarose. His-ubiquitinated proteins were eluted, resolved by SDS-PAGE and immunoblotted with the indicated antibodies. The relative abuncance of HA signal in the Ni-pull down normalized to the His-Ub signal is indicated. See also Fig. S3c (**h**) Mutations of Chk1-mediated phosphorylation sites in Cdh1 increases the stability of Cdh1. 293T cells were transfected with the indicated HA-Cdh1 constructs together with Flag-βTRCP1 and Myc-Chk1^L449R^ (where indicated). Cells were treated with 50 µg/ml cycloheximide (CHX). At the indicated time points, whole-cell lysates were prepared for immunoblot analysis. The intensities of Cdh1 bands were normalized to actin, then normalized to the *t* = 0 time point. The plots represent the relative fraction of indicated Cdh1 protein at indicated time point after adding CHX. Data are represented as mean ± SD, *n* = 3 biological replicates. See also Fig. S3d.

Recognition of Cdh1 by βTRCP1 is mediated via the DDGNDVS sequence, which closely resembles the canonical DpSGx(2-4)pS degron sequence (where pS designates phosphorylated Ser)^8,33,53–55^. Notably, residues flanking the non-canonical degron were implicated in efficient binding of Cdh1 by βTRCP1^33^. Given that Chk1 promotes Cdh1 degradation in response to DNA damage^34^ and that Chk1 positively impacts the binding between Cdh1 and βTRCP1 both in vitro and in vivo, we explored the Chk1 phosphorylation event on Cdh1 more closely. We first confirmed that Cdh1 is directly phosphorylated by Chk1 in vitro (Fig. 3a). The phosphorylated GST-Cdh1 was not detected in the presence of Chk1i or after phosphatase treatment (Fig. 3a).

Intriguingly, S138, which was previously implicated in recognition of Cdh1 by βTRCP1, matches the minimal Chk1 consensus R/K-x-x-S/T (Fig. 3b), suggesting that Chk1 may promote βTRCP1 binding by phosphorylating this site. However, although phosphorylation of S138 and S137 could be identified by mass-spectrometry analysis, these residues are not an efficient sites for Chk1-mediated phosphorylation in vitro (not shown). In contrast, peptides containing phosphorylation at S131 and S133, S131 or S133, and S172 were abundant with ratios of modified to unmodified peptides of ~1, 20, and 34, respectively (Figs. 3b and S2). S172 resembles the Chk1 consensus while S131 and S133 do not. Notably, multiple examples of Chk1 substrates with non-conventional phosphorylation sites exist (www.phosphosite.org). By incubating recombinant Cdh1 in extracts derived from G1 cells we have previously demonstrated that S133 and S172 are phosphorylated by cellular kinases^56^. Here, we demonstrate that acute treatment of extracts with Chk1i prior to incubation of recombinant GST-Cdh1 diminished the phosphorylation of S133 by 50% and S172 by 30% (Supplementary Fig. S3b), consistent with the reduced Cdh1-βTRCP1 interaction observed upon inhibiting Chk1 in extracts (Fig. 2a). These data indicate that Cdh1 is a substrate of Chk1.

We next examined the possible involvement of these sites in βTRCP1 binding by introducing serine-to-alanine mutations. Given the previous demonstration of S138 in regulating Cdh1 degradation we included S137 and S138 mutants in our studies^33^. The interaction between transfected Cdh1 and endogenous βTRCP1 was reduced by serine-to-alanine replacements of the Chk1 phosphorylation sites that we identified in Cdh1 as well as the by the loss of degron-adjacent phosphorylation sites SS137/138AA (Fig. 3c)^33^. We next examined the impact of Chk1 activity on the Cdh1-βTRCP1 interaction in the absence of additional replication stress responses. Thus, we utilized a constitutively active Chk1 mutant, Chk1^L449R^^57^. Cdh1 interacts with Chk1^L449R^ when co-expressed in 293T cells (Fig. 3d). In agreement with the role for Chk1 in the Cdh1-βTRCP1 interaction, co-expression of Chk1^L449R^ with HA-Cdh1 and Flag-βTRCP1 led to increased Cdh1-βTRCP1 binding (Fig. S3a) in vivo. These data indicate that Cdh1 phosphorylation by Chk1 is a key step in the induction of βTRCP1 binding.

### Chk1-mediated phosphorylation sites regulate Cdh1 stability and turnover

Our results thus far indicate that Chk1 contributes to recognition of Cdh1 by SCF^βTRCP1^, which triggers Cdh1 degradation in late G1 and early S-phase. We thus examined the impact of Chk1-mediated Cdh1 phosphorylation on Cdh1 protein dynamics at G1/S. Consistent with our βTRCP1-binding data, mutation of Chk1 phosphorylation sites in Cdh1 or S137/S138, delayed the degradation of transfected HA-Cdh1 compared to the wild type after release from mitosis (Fig. 3e). Notably, although the S137/S138 did not stabilize Cdh1 to the same extent as the other mutations, the stabilization was evident and reproducible, particularly at 9 h after nocodazole release (*p* = 0.051). To highlight the specific function of Chk1-mediated Cdh1 phosphorylation on Cdh1 degradation as cells progressed to G1/S, HeLa cells were treated with the Chk1 inhibitor CHIR-124 4 h after release from a mitotic block. The loss of endogenous Cdh1 protein as cells progressed toward the G1/S transisiton was muted in the presence of Chk1i (Fig. 3f). This observation mimics the requirement for βTRCP1 in degradation of Cdh1 at G1/S^33^ and further indicates that Chk1-mediated phosphorylation promotes Cdh1 degradation in late G1, early S-phase.

To examine the possible involvement of Chk1 sites in βTRCP1-dependent ubiquitination of Cdh1, in vivo ubiquitination assays were carried out. We first determined that Chk1^L449R^ was able to stimulate ubiquitination of Cdh1 in the presence of βTRCP1 (Fig. S3c). We next performed analyses with phosphorylation site mutants of Cdh1. Ubiquitination was reduced with the alanine mutants of Cdh1 compared to wild type even in the presence of the consitutively active Chk1^L449R^ mutant (Fig. 3g). Similarly, co-expression of βTRCP1 and Chk1^L449R^ reduced the stability of wild type Cdh1 in the presence of cycloheximide whereas S131/133A, S137/138A and S172A mutant Cdh1 proteins were not impacted by the presence of Chk1^L449R^ (Figs. 3h and S3c). Overall, these data established that Chk1-dependent phosphorylation of Cdh1 is important for its recognition by βTRCP1 and subsequent ubiquitination. These data also support our hypothesis that multiple kinases work together to regulate Cdh1 levels for proper cell-cycle transition.

### Maintaining Chk1 activity inhibits the accumulation of Cdh1 through S/G2 phase

**Fig. 4:**
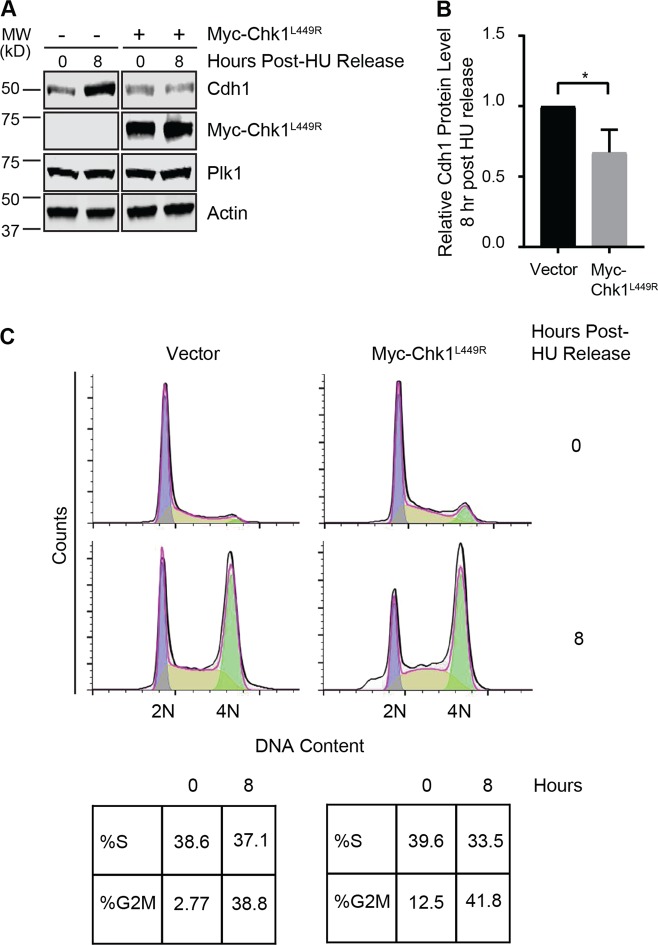
Presence of constitutively active Chk1 inhibits the accumulation of Cdh1 through S/G2 phase. **a** 293T cells were transfected with Myc-Chk1^L449R^, treated with HU (2 mM) for 20 h and released into fresh media. At the indicated time points, cells were harvested to prepare the whole-cell lysates for immunoblot analysis. **b** Quantification of band intensities as in (**1e**). The intensities of Cdh1 were normalized to actin and then further normalized to vector control. Data are represented as mean ± SD, *p < 0.05 by unpaired *t*-test, *n* = 4. **c** Cell-cycle progression was monitored by flow cytometry. The experimental set up was as described as in (**a**).

Our data indicate that Chk1, in addition to Cyclin A and Plk1, promotes SCF^βTRCP1^-mediated Cdh1 degradation at G1/S. In contrast to high levels of Cyclin A-Cdk2 and Plk1 activities, multiple mechanisms cooperate to attenuate Chk1 activity in G2^40,45,58–63^. We therefore hypothesized that in G2 the absence of Chk1 activity facilitates Cdh1 stability and accumulation. To test this possiblity, we expressed constitutively active Chk1^L449R^ in 293T cells synchronized in S-phase by HU treatment. Indeed, constitutive Chk1 activity in cells reduced endogenous Cdh1 accumulation through S/G2 upon release from HU (Fig. 4a, b). The cells progressed through S/G2 phase normally as confirmed by flow cytometry analysis (Fig. 4c). These data support down-regulation of Chk1 activity as a mechanism that allows for Cdh1 stability in G2.

### Chk1-mediated phosphorylation of Cdh1 ensures proper cell-cycle progression

**Fig. 5:**
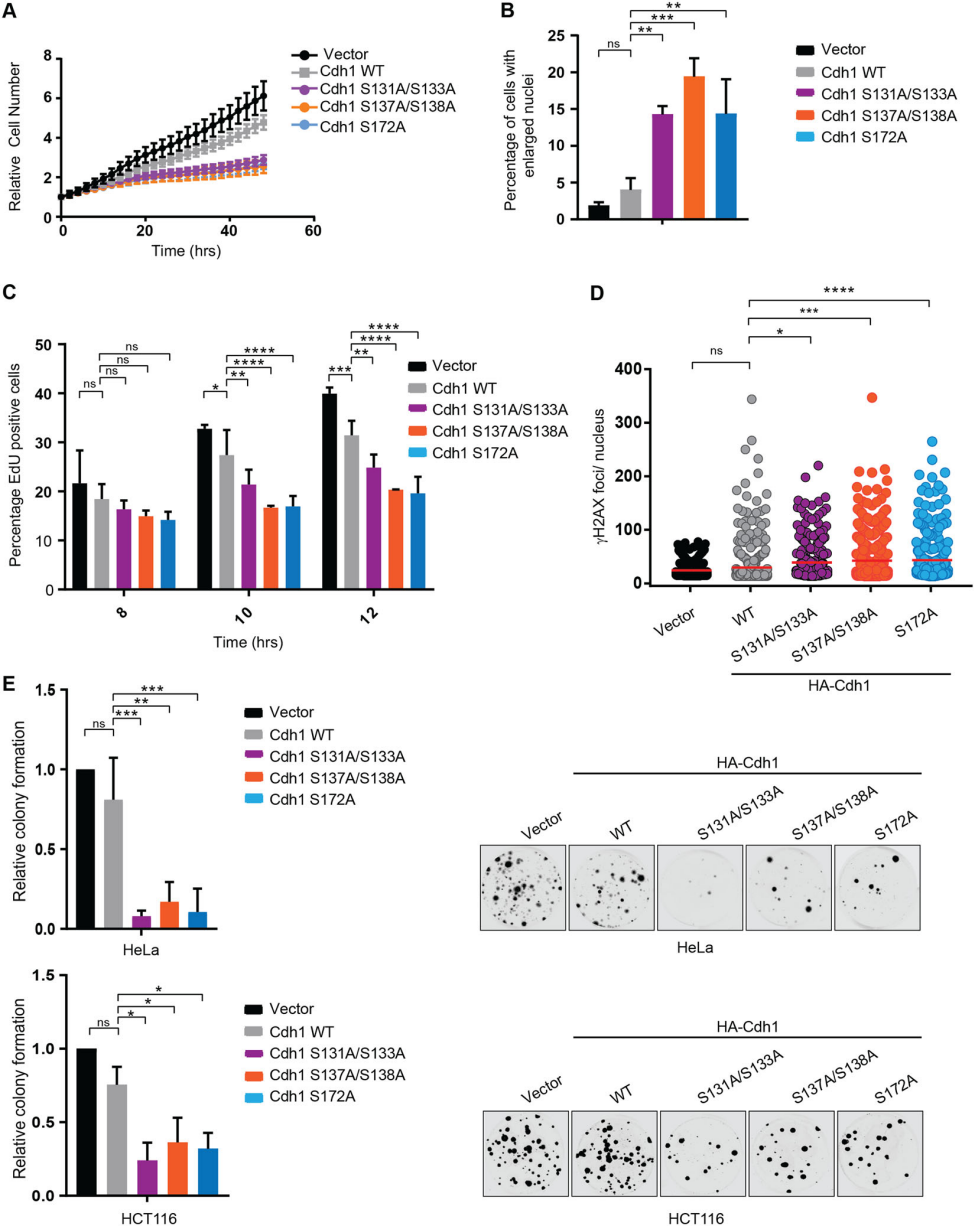
Chk1-mediated phosphorylation of Cdh1 ensures proper cell-cycle progression. **a** Mutation of the Chk1-mediated Cdh1 phosphorylation sites inhibits proliferation. HeLa cells were transfected with histone H2B-GFP and either vector or the indicated Cdh1 constructs. After 24 h, proliferation of the GFP-positive cells was analyzed over time. **b** Mutation of the Chk1-mediated Cdh1 phosphorylation sites promotes aberrant nuclear size. HeLa cells were transfected as in A. After 48 h, the percent of GFP-positive cells with enlarged nuclei was determined. Data (**a**, **b**) are represented as mean ± SD, *n* = 3 biological replicates **p < 0.01, ***p < 0.001 were calculated with 1-way ANOVA with Sidak’s post-test. **c** Mutation of the Chk1-mediated Cdh1 phosphorylation sites delays S-phase entry. Hela cells (as in **a**) were synchronized in mitosis with a thymidine-nocodazole block following transfection with the indicated Cdh1 constructs. After release from nocodazole, cells were pulsed with EdU for 15 min, at the indicated time points fixed and analyzed for EdU incorporation. The plot represents the percent of GFP-positive, EdU-positive nuclei. Data are represented as mean ± SD, *n* = 3 biological replicates and *p < 0.05, **p < 0.01, ***p < 0.001, ****p < 0.0001 were calculated with 2-way ANOVA with Dunnet’s post-test. **d** Mutation of the Chk1-mediated phosphorylation sites in Cdh1 induces DNA damage. The number of γH2AX foci in cells. U2OS cells as in (**a**) were analyzed for γH2AX foci. The graph shows the number of γH2AX foci per GFP-positive nucleus. Mean is indicated by the red line. *n* > 275; *p < 0.05, ***i < 0.001, ****p < 0.0001 were calculated with 1-way ANOVA with Dunnet’s post-test. **e** Effects of non-degradable Cdh1 on colony-forming capacity of HeLa and HCT116 cells. Cells were transfected with the indicated Cdh1 constructs. The graph represents the relative fraction of number of colonies transfected with each indicated Cdh1 constructs and normalized to vector control. *n* = 3 biological replicates (with three technical replicates per biological replicate), error bars represents standard deviation and *p < 0.05, **p < 0.01, ***p < 0.001 were calculated with 1-way ANOVA with Sidak’s post-test. Representative images of colony formation following each treatment are shown for both HeLa and HCT116 cells.

Given that mutation of the Chk1 phosphorylation sites of Cdh1 extended the half-life of Cdh1 and considering the importance of Cdh1 inactivation for S-phase entry, we next investigated the physiological significance of Chk1-mediated Cdh1 phosphorylation on cell growth. HeLa cells were transfected with both wild type and Cdh1 phosphosite mutants along with H2B-GFP and proliferation was monitored in real time. Cells expressing Cdh1 phosphosite mutants showed a reduction in proliferation in comparison to both control and wild type-expressing cells (Fig. 5a). In addition, the phosphosite mutants induced an increased number of cells with enlarged nuclei relative to wild type and control cells (Figs. 5b and S4a), consistent with the DNA re-replication caused by aberrantly increased APC/C^Cdh1^ activity^19,24,64–66^. To specifically address the role of Chk1-mediated phosphorylation of Cdh1 on S-phase entry, we monitored the progression of cells through G1 and into S-phase following release from a mitotic arrest. Cells were pulse labeled with EdU to identify replicating cells 8, 10, and 12 h after release from the mitotic block. Cells expressing Cdh1 phosphorylation site mutants exhibit delayed S-phase entry (EdU+ cells) compared to vector control or wild type Cdh1-expressing cells (Figs. 5c and S4b).

As aberrant APC/C^Cdh1^ activity is associated with replication stress, cells were examined for foci of DNA damage markers γH2AX and 53BP1. Cells expressing Cdh1 phosphosite mutants showed increased numbers of these foci indicating a higher level of DNA damage (Figs. 5d, S4c, and S5). In addition, stabilization of Cdh1 lowered clonegenicity for both HeLa and HCT116 cells (Fig. 5e). Our data demonstrate that Chk1 phosphorylation makes a significant contibution to SCF^βTRCP1^-mediated down-regulation of Cdh1 for timely S phase entry and enhanced maintenance of genomic stability.

### Chk1-mediated phosphorylation of Cdh1 destabilizes the Interaction between Cdh1 and the APC/C

**Fig. 6:**
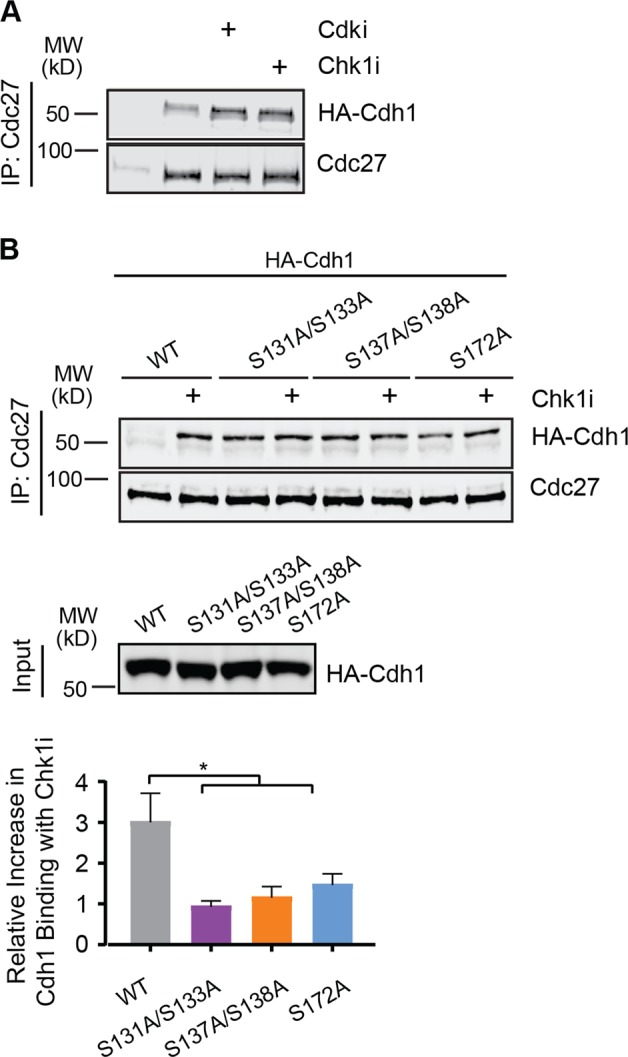
Chk1-mediated phosphorylation of Cdh1 destabilizes the Cdh1–APC/C interaction. **a** In vitro binding of Cdh1 to the APC/C in G1/S extracts from HU-arrested HeLa cells with or without Cdki (purvalanol) or Chk1i. Immunoblots represent both immunoprecipitated HA-Cdh1 and the APC/C subunit Cdc27. **b** In vitro binding of wild-type or mutant Cdh1 proteins to the APC/C in G1/S extracts from HU arrested HeLa cells with or without Chk1i. Immunoblots represent both immunoprecipitated HA-Cdh1 constructs and the APC/C subunit Cdc27 with the relative inputs of the Cdh1 constructs (*n* = 3). Data represent mean and SEM. Significance was determined by one-way ANOVA with Holm-Sidak post-test, *p < 0.05.

The finding that transfection of WT Cdh1 elicits only modest biological effects was surprising given that these cells contain a readily detectable amount of Cdh1 compared to control cells and suggests that cells were able to compensate for the additional WT Cdh1 but cannot accommodate the mutant proteins. We thus considered the possibility that the Chk1 phosphorylation sites may regulate the binding of APC/C and Cdh1. The APC/C–Cdh1 interaction is known to be regulated by the phosphorylation status of Cdh1^9,24–27,30^. The Chk1 phosphorylation sites on Cdh1 are proximal to the APC/C binding interface of the Cdh1 C-box and KLLR motifs and could potentially influence APC/C–Cdh1 interactions^29^. To test this idea, in vitro translated Cdh1 proteins were incubated with G1/S HeLa cell extracts treated with or without Chk1i. As a control we included the Cdk inhibitor purvalanol as Cdk activity is known to perturb APC/C–Cdh1 binding in these extracts. Cdh1–APC/C binding was examined by co-immunoprecipitation of Cdh1 with the APC/C. As expected, Cdh1 showed minimal binding to APC/C in these extracts^19,24,28^. However, APC/C–Cdh1 binding was increased upon inhibition of either Chk1 or Cdks (Fig. 6a). We then investigated the impact of the Cdh1 phosphosite mutants on the APC/C interaction. Cdh1 phosphosite mutants showed higher basal APC/C binding that was not increased by Chk1i (Fig. 6b). These data suggest that Chk1 negatively regulates APC/C^Cdh1^ activity by both promoting Cdh1 destruction and by destabilizing its association with the APC/C.

## Discussion

Overall, our study has identified Cdh1 as a previously unknown substrate of the Chk1 kinase, which provides new insight into the regulation of Cdh1 by both Chk1 and SCF^βTRCP^. First, we have identified Chk1 phosphorylation of Cdh1 in the regions flanking the SCF^βTRCP^-recognized phosphodegron as an important facet in the efficient recognition of Cdh1 by the ubiquitin ligase. Second, we have demonstrated that phosphorylation of Cdh1 by Chk1 perturbs the ability of the activator to bind APC/C.

Consistent with a role for Chk1 in Cdh1 degradation^34^, exposure to etoposide or aphidicolin appears to decrease Cdh1 stability in a βTRCP-dependent manner^33^, while UV radiation, another potent inducer of Chk1 activity, triggers Cdh1 degradation^43^. Although the role of Chk1 and SCF^βTRCP^ were not formally examined in the latter study, the degron-containing region of Cdh1 identified in these studies contains the SCF^βTRCP^ phosphodegron and the Chk1 phosphorylation sites, supporting a role for Chk1 in these events. Plk1 is responsible for phosphorylation of the core degron, DDGNDVS, at S146. Given that Plk1 activity increases during G2 it was not clear how Cdh1 becomes stable at this point in the cell cycle. However, phosphorylation of the region flanking the core degron, including S138, is implicated in SCF^βTRCP^-Cdh1 binding suggesting that an additional kinase(s) is required for efficient targeting of Cdh1 by SCF^βTRCP^^33^. The involvement of an S-phase active kinase (e.g., Chk1) in efficient Cdh1-SCF^βTRCP^ binding would provide a mechanism that allows Cdh1 protein to accumulate during G2 even as Plk1 activity rises^67^. Indeed our data suggest that down-regulation of Chk1 activity promotes Cdh1 accumulation in G2.

From a mechanistic standpoint, the region surrounding S138 resembles the Chk1 consensus site and Chk1 has recently been demonstrated to promote Cdh1 degradation upon replication stress^34^. However, although mutation of S137 and 138 rendered Cdh1 resistant to Chk1-enhanced degradation, we did not obtain evidence for robust direct phosphorylation of these residues by Chk1. We interpret this result to indicate that Chk1 may indirectly regulate the phosphorylation of these residues as has been demonstrated for Cdc25A^68^. However, we cannot exclude the possibility that S137, S138 of our recombinant Cdh1 is not available to the kinase.

In contrast to S137, S138, Chk1 efficiently catalyzed the direct phosphorylation of containing S131, S133, and S172 and mutation of these sites abolished the ability of Chk1 to promote Cdh1 degradation. These sites are poorly characterized, with a single reported identification for pS131 and pS133 and no previous reports of pS172 in proteomic databases. Phosphorylation at S131/S133 is well positioned to contribute to the recognition of Cdh1 by βTRCP as supported by the ability of Chk1 to promote binding between Cdh1 and βTRCP in vitro. The contribution of S172 phosphorylation is less clear as the distance from the phosphodegron would suggest that this site is less likely to contribute to βTRCP-binding. From a structural standpoint, S172 appears to reside at the C-terminal end of a linker between the APC/C interacting KLLR motif and the WD40 domain and may pack against the WD40 propeller^29^. Thus, phosphorylation of this residue could impact the Cdh1 structure and/or APC/C–Cdh1 binding. Similarly, S131, S133, S137, and S138 lie within a region that is also involved in phospho-regulation of APC/C–Cdh1 binding.

Indeed, our data indicate that Chk1 regulates APC/C–Cdh1 binding and support an important role for this function. Whereas the phosphosite mutant Cdh1 proteins exhibit increased stability and inducibly-expressed proteins remain at higher levels in late G1 when expression is limited by doxycycline-withdrawal, constitutively expressed proteins do not show dramatically different steady state levels and express a significant amount of wild-type protein in late G1, although less than the mutant proteins (AET and MKS, unpublished observations). Given that constitutive expression of the wild-type protein has little impact on S-phase entry, DNA damage, or cell growth, these observations suggest that perhaps displacing Cdh1 from APC/C is a key function of Chk1-mediated phosphorylation of Cdh1, as has been previously reported for Cdk2^9,24–30,64^, at least under non-perturbed conditions. These findings are in line with studies in fission yeast where the replication stress checkpoint effector, Cds1, negatively regulates APC/C activity by phosphorylating the Cdh1 homolog Ste9 and preventing its interaction with the holoenzyme^69^. Intriguingly, we identified increased phosphorylation of both S133 and S172 in glioblastoma cancer stem cells, which have attenuated APC/C^Cdh1^ activity that is associated with both diminished APC/C–Cdh1 interactions and lower Cdh1 protein levels^56,70^. Notably, glioblastoma stem cells are also known to have high basal Chk1 activity^71^. Whether displacing Cdh1 from APC/C also facilitates degradation by promoting availability to kinases (e.g, Plk1) and/or to βTRCP or whether displacent or degradation is the critical function of these phosphorylation events is not clear and will require further research to discriminate between these possibilities.

Biologically, as rising Plk1 activity in G2 contributes to the down-regulation of Chk1 activity by multiple ligases, which would both weaken recognition of Cdh1 by βTRCP and facilitate APC/C–Cdh1 binding to prevent further Chk1. In addition, as enhanced Chk1 activation leads to early S-phase entry, our data may suggest that interplay between Chk1 and Cdh1 sharpens the G1/S and S/G2 transitions^37,58–63^. Similarly, our data may provide new insight into the relationship between APC/C^Cdh1^ and SCF^Cyclin F^ in these transitions as well. Treatment of Cyclin F knockout cells with Chk1i induces replication catastrophe^72^. Given that Cdh1 is an SCF^Cyclin F^ substrate, it is possible that Chk1 is required to allow these cells to cope with elevated Cdh1 levels, which would contribute to the observed replication defects.

Given that loss of APC/C^Cdh1^ activity is associated with genomic instability and tumorigenesis it is tempting to speculate that upregulation of Claspin, Chk1, Cyclin A, and Plk1 may contribute to genomic instability and cancer, in part, via antagonism of APC/C^Cdh1^. In contrast, while multiple mutations in *FZR1*, the gene encoding Cdh1, across several tumor types cluster in the degron region of the protein, the majority of these mutations (e.g., D140N, D144N, within the core degron) would seem likely to antagonize down-regulation of Cdh1 rather than promote it (www.cbioportal.org). Our observations of inefficient replication initiation, increased DNA damage, and evidence of genomic instability in the form of DNA re-replication in cells that failed to down-regulate Cdh1 is in direct line with previous studies showing that down-regulation of Cdh1 at the G1/S transition and during S-phase, particularly in the presence of replication stress, is critical for cellular viability and genomic stability. Notably, the more conservative mutations in non-phosphorylated sites within this region may have a less severe impact on the degradation of Cdh1 than ablation of Cdh1 phosphorylation sites used in this and previous studies. Thus, we postulate that future examination of these tumor-derived mutations will reveal increased genomic instability, but at a level compatible with viability, and provide further evidence that altered regulation of APC/C^Cdh1^, either positive or negative, has pathophysiological consequences. Finally, while our data highlight the importance of these Chk1-responsive phosphorylation sites in the regulation of Cdh1 they also leave open the possibility that additional kinases may also contribute to Cdh1 regulation via these sites, perhaps indicating that differnet pools of Cdh1 may be regulated via different pathways that converge on this degron.

## Materials and methods

### Mammalian cell culture, synchronization, and drug treatments

HeLa, 293T and HCT116 cells were obtained from ATCC and maintained in DMEM complete medium (Corning) supplemented with 10% fetal bovine serum (FBS; Seradigm). Hela cells were synchronized in S-phase by double thymidine block with 2 mM thymidine and transfection between the blocks followed by treatment with 50 ng/ml nocodazole to arrest cells in mitosis. The cells were then washed two times with fresh DMEM complete medium and replated into nocodazole-free fresh medium. Chk1 inhibitor was added where indicated 3 h post mitotic release. For USP37 siRNA treatment, HeLa cells were transfected with RNAiMAX (Invitrogen) per the manufacturer’s instructions between double thymidine blocks. 293T cells were arrested at G1/S phase in 2 mM hydroxyurea for 16 h and washed with PBS and released into fresh DMEM complete medium. Plasmid transfections were done with TransIT-LT1 (Mirus Bio) per the manufacturer’s instructions. Where indicated, cells were treated with 500 nM CHIR-124 (Selleckchem), 1 µM AZD7762 (Selleckchem), 200 nM BI2536 and 10 µM MG132 (Boston Biochem). Cycloheximide (CHX) assay was performed as described previously.

Immunofluorescence, microscopy, and flow cytometry were performed as previously described^73^. Detection of DNA synthesis in proliferating cells was determined based on the incorporation of 5-ethynyl-2′-deoxyuridine (EdU; Thermo Fisher Scientific) and its subsequent detection by a fluorescent azide through a Cu(I)-catalyzed [3 + 2] cycloaddition reaction (“click” chemistry) per the manufacturer’s instructions. In brief, HeLa cells were transfected with different Cdh1 constructs and histone H2B-GFP as a tracer and synchronized in S-phase with double treatment with 2 mM thymidine and then arrested at G2/M phase in 50 ng/ml Nocodazole for 16 h treatment. The cells were then washed two times with fresh DMEM complete medium and replated into nocodazole-free fresh medium and pulsed for 15 min with 10 µM EdU (Thermo Scientific) at 8 h, 10 h, and 12 h time points and fixed in 3.7% formaldehyde, and washed in PBS prior to EdU labeling by click chemistry. Cell populations were imaged with the IncuCyte ZOOM and the fraction of EdU-positive transfected (GFP-positive) cells was determined using the coincident analysis application within the IncuCyte software. For detection of DNA damage U2OS cells were seeded on glass coverslips and transfected with different Cdh1 constructs and histone H2B-GFP as a tracer. After 48 h cells were fixed and permeablized with 0.5% Triton X-100 in PBS, washed and then blocked for 30 min at room temperature with 5% BSA in PBS. Cells were incubated with antibodies (1:500) in 5% BSA in PBST for 1 h at room temperature. After washing the cells were incubated with Alexafluor secondary antibodies (1:500) in 5% BSA in PBST for 30 min at room temperature. DNA was counterstained with 1 µg/mL Hoechst 33342 and mounted with Fluorimount G (Southern Biotech). Cells were imaged using a Leica DM5500B fluorescent microscope as described previously^73^. Images were analyzed and foci quantified with Cell Profiler software^74^.

### Plasmids and recombinant proteins and siRNA

HA-Cdh1 and different mutants of HA-Cdh1, cloned into pcDNA 3.1 were obtained from GenScript. The Cdh1 cDNA was amplified with PCR and the PCR products were subcloned into pCS2+ and pGEX-4T-1 vectors. Myc-Chk1, Myc-βTRCP1, Flag- βTRCP1, and HA-Plk1 were generated as described previously^73^. cDNA for Chk1 was a gift from Youwei Zhang and was subcloned into modified pCS2 vectors using Gateway cloning. Q5 mutagenesis was used to introduce Chk1^L449R^ mutation. Recombinant and in vitro translated proteins were produced as described^73^. Chk1 small interfering RNA (siRNA) oligonucleotide sequences were purchased from Dharmacon (ON-TARGET*plus* siRNA HUMAN CHEK1).

### Antibodies

The following commercial antibodies, and the indicated concentrations, were used in this study. C-Myc (#E0115; 1:1000), Chk1 (G-4) (#H2714; 1:1000) and GST (Z-5) (#K0713; 1:1000) were purchased from Santa Cruz Biotechnology. M2 anti Flag Mouse antibody (#SLBT7654; 1:5000), cdc27 (AF3.1) (1:1000) and Actin (#087M4850; 1:10,000) were purchased from Sigma. Cdh1 (#CC43-100UG; 1:500) was purchased from Calbiochem. Cyclin A2 (BF683) (#6; 1:1000), βTRCP1 (D13F10) (1:1000) and Phospho-Chk1Ser345 (133D3) (#15; 1:1000) were obtained from Cell Signaling. HA (#SJ254200; 1:1000) antibody was purchased from Biolegend. Plk1 (3F8) (#06050819; 1:500) was obtained from Enzo Life Sciences. HA antibody (HA.C5 #18181) (1:1000) was purchased from Abcam. Secondary antibodies for western blotting were purchased from LI-COR Biosciences. Anti-phospho-Histone H2AX (clone JBW301) (#2977883, 1:500) was purchased from EMD Millipore Corp. Alexa546-conjugated antibodies (#A11030) for immunofluorescence were purchased from Invitrogen.

### Western blotting and immunoprecipitation

Either HA-tagged Cdh1 and Myc-tagged Chk1 mutant or HA-tagged Cdh1 (or mutants) and Flag- βTRCP1were expressed where indicated in 293T cells for 30 h. Cells were treated with MG-132 (10 µM for 5 h) prior to lysis. Cell extracts were generated in EBC buffer, 50 mM Tris (pH 8.0), 120 mM NaCl, 0.5% NP40, 1 mM DTT, and protease and phosphatase inhibitors tablets (Thermo Fisher Scientific).For immunoprecipitation, equal amounts of cell lysates were incubated with the indicated antibodies conjugated to protein G beads (Invitrogen) or anti-HA beads (15 µl per IP, Thermo Scientific) respectively from 4 h to overnight at 4 ***°***C. The beads were then washed with EBC buffer including inhibitors. Binding to immobilized GST proteins was performed as described previously^33^. Immunoprecipitation samples or equal amount of whole-cell lysates were resolved by SDS-PAGE, transferred to PVDF membranes (Milipore) probed with the indicated antibodies, and visualized with the LiCor Odyssey infrared imaging system.

### In vitro kinase assay

Five microgram indicated GST-Cdh1 fusion proteins was incubated with kinase reaction buffer (50 mM Tris pH 7.4, 10 mM MgCl_2_, 1 mM DTT, phosphatase inhibitors and 200 µM ATP) and 100 ng of Chk1 (Sigma) at 30 ***°***C for 45 min. To inhibit Chk1, 500 nM CHIR-124 was included in the reaction buffer. Phosphorylated samples were precipitated on the glutathione beads (Life Technologies) and resolved by SDS-PAGE. For phosphatase treatments, bead-bound GST-Cdh1 was incubated with 200U Lamda Protein Phosphatase (NEB) as per the vendor’s protocol for 30 min at 30 ***°***C. Phosphorylation of GST-Cdh1 was detected by pIMAGO phosphoprotein detection kit (Tymora Chemicals). For mass-spectrometry analysis, the proteins were resolved on SDS-PAGE and visualized with Gelcode Blue (Pierce).

### In vitro Cdh1 binding assay

Kinase reactions were perforemed as above in the with or without Chk1 inhibitor CHIR-124 (500 nM). Phosphorylated samples were precipitated on glutathione beads (Life Technologies). In vitro translated HA-βTRCP1 (T_N_T quick coupled Transcription/Translation system, Promega) was incubated with the bead-bound GST-Cdh1 for 1 h at 4 ***°***C. Beads were then washed and proteins resolved by SDS-PAGE and analyzed by western blotting as above.

### Extract-mediated phosphorylation and binding assays

HeLa cells were synchronized and harvested in G1/S boundary, after a 2 mM hydroxyurea (HU) treatment for 16 h. Extracts were then prepared by resuspension in extract buffer (20 mM Tris-HCl, pH 7.2, 2 mM DTT, 0.25 mM EDTA, 5 mM KCl, 5 mM MgCl2) followed by two rounds of freeze-thaw and passage through a needle. Extracts were supplemented with ATP and an energy regenerating system. For GST-Cdh1 binding, GST-Cdh1 was incubated in extract in presence of Chk1 inhibitor CHIR-124 (500 nM), where indicated, for 1 h at 30 °C. Binding to in vitro translated HA-βTRCP1 was performed and analyzed as above. For mass-spectrometry analysis, GST-Cdh1 was resolved on SDS-PAGE and visualized with Gelcode Blue. For Cdc27 binding, in vitro translated HA-Cdh1 proteins (as above) were then incubated in extract in presence of Chk1 inhibitor CHIR-124 (500 nM), where indicated, for 1 h at 30 °C. Cdc27, and interacting proteins, were then immnoprcipitaed using anti-Cdc27 antibody (AF3.1, Sigma) bound to protein G beads (Invitrogen) overnight at 4 °C. After washing, the proteins were resolved on SDS-PAGE and analyzed by western blotting as above.

### Mass spectrometry

Protein bands derived from phosphorylated GST-Cdh1, prepared by in vitro kinase or extract-mediated phosphorylation reactions, as above, were reduced with DTT, alkylated with iodoacetamide, and digested with trypsin or chymotrypsin, extracted in 50% acetonitrile; 5% formic acid. After evaporation, peptides were resuspended in 1% acetic acid and analyzed on a Thermo Scientific Ultimate 3000 UHPLC + Orbitrap Elite hybrid Mass spectrometer. Dionex 15 cm ×75 μm id Acclaim Pepmap C18, 2μm, 100 Å reversed phase capillary chromatography column was utilized. Peptides were eluted from the column by an acetonitrile/0.1% formic acid gradient. The digest was analyzed using the data dependent multitask capability of the instrument acquiring full scan mass spectra to determine peptide molecular weights and product ion spectra to determine amino acid sequence in successive instrument scans.

The data were analyzed by using all collisionally induced dissociation (CID) collected in the experiment to search the human UniProtKB database with the search programs Sequest and Mascot and more specifically against the sequence of GST-Cdh1 using the program Sequest. Protein and peptide validations were performed with the Program Scaffold.

### In vivo ubiquitination

The in vivo ubiquitination assays were performed as described previously^33,73^. Briefly, 293T cells were transfected with the constructs encoding HA-Cdh1 or HA-Cdh1 mutants, His-ubiquitin, Flag- βTRCP1 and Myc-Chk1^L449R^ respectively. After a treatment with 20 µM MG132 for 5 h, the cells were lysed with denaturing buffer (6 M guanidiniu-HCl, 0.1 M Na2HPO4/ NaH2PO4, 10 mM Tris-HCl pH 8.0, 10 mM Beta-Mercaptoethanol and 5 mM imidazole pH8.0), followed by sonication. After centrifugation, the lysates were collected and incubate with Ni-NTA agarose beads (QIAGEN) for 4 h. His-ubiquitinated proteins were washed three times with denaturing buffer (8 M urea, 0.1 M Na2HPO4/ NaH2PO4, 10 mM Tris-HCl pH 6.3, 10 mM Beta-Mercaptoethanol and 0.2% or 0.1% triton-X-100) and eluted with elution buffer (150 mM Tris-HCl pH 6.7, 200 mM imidazole), resolved by SDS-PAGE and immunoblotted with the indicated antibodies.

### Clonogenic survival assay

HeLa and HCT116 cells were transfected with HA-Cdh1 (wild type or mutants) and pLKO.1 plasmid. 20 h post-transfection, cells were split into 6 well plates. Cells were then treated with 2 mM Hydroxyurea for 16 h and released into fresh DMEM with puromycin (1 µg/ml) followed by two washes with PBS. The cells were selected for 72 h. After 8 days, the colonies were stained on the plates with crystal violet and counted. The images of the plates were taken in Li-COR and colonies were counted manually.

### IncuCyte proliferation assay for live-cell analysis

Hela cells were transfected with the indicated DNAs and histone H2B-GFP as a tracer. 48 h later the cells were placed in the IncuCyte Zoom System (Essen BioScience). Images were collected at 10× every 4 h for 3 days. Proliferation of transfected cells was analyzed by quantifying GFP-positive nuclei in the Proliferation-Cell Count application of the IncuCyte software. Nuclear size was also determined using this application. Large nuclei were identified as having a size greater than or equal the largest nuclei frequently observed in control populations.

### Statistical analysis

Statistical analyses were performed on individual experiments, as indicated, with the GraphPad Prism 8 Software using an unpaired *t*-test, equal variance, for comparisons between two groups or a 1- or 2-way ANOVA with Holm-Sidak’s or Dunnet’s post-test, respectively, where for more than two conditions are compared (GraphPad Software, Inc.). Sample sizes and specific tests are indicated in the figure legends. A *p*-value of <0.5 was considered significant.

## Supplementary information

Supplemental Figure Legends

Figure S1

Figure S2

Figure S3

Figure S4

Figure S5
