# Supplementary material for: Chk1-mediated phosphorylation of Cdh1 promotes the SCFβTRCP-dependent degradation of Cdh1 during S-phase and efficient cell-cycle progression
Source: Cell Death Dis. 2020 Apr 28;11(4):298. doi: 10.1038/s41419-020-2493-1 (PMC7188793; doi:10.1038/s41419-020-2493-1)
Supplement: Supplementary file 1 — Supplemental Figure Legends [file 41419_2020_2493_MOESM1_ESM.docx]

**Figure S1: Inhibition of Chk1 Enhances Stability of Endogenous Cdh1.**

**(A)**HeLa cells were treated Chk1 inhibitor, CHIR-124 (500 nM) (where indicated) for 4 hr before adding 50 µg/ml cycloheximide (CHX) and HU. At the indicated time points, whole-cell lysates were prepared for immunoblot analysis.

**(B)** 293T cells were transfected with wild type HA-Cdh1 construct and treated with Chk1 inhibitor, AZD7762 (1 µM) ) where indicated and the proteasome inhibitor MG132 (10 µM) for 5 hr before immunoprecipitation to detect the interaction between HA-Cdh1 and endogenous βTRCP1 proteins.

**Figure S2: Mass spectrometry analysis of Chk1 mediated Cdh1 phosphorylation.** Chymotryptic digests were performed on the Cdh1 protein in order to identify sites of phosphorylation.

**(A)** The MS/MS spectra of a doubly charged SLSTKRSSPDDGNDVSPY + PO3 peptide was identified with a m/z ratio of 1002.9299 Da. The site of phosphorylation was identified to be either S131 or S133 by the mass of the y15 ion.

**(B)** The MS/MS spectra of a doubly charged SLSTKRSSPDDGNDVSPY + 2PO_3_ peptide was identified with a m/z ratio of 1042.9131 Da. The sites of phosphorylation was identified to be S131 and S133 by the mass of the b_3_ ion.

**(C)** The MS/MS spectra of a doubly charged KIpSKIPF peptide was identified with a m/z ratio of 456.7511 Da. The site of phosphorylation was identified to be S172 by the mass difference between the y_5_ and y_4_ ions

**Figure S3: Phosphorylation of Cdh1 by Chk1 Creates a Phosphodegron Recognized by SCF^βTRCP1^, Related to Fig. 3.**

**(A)** Constitutively active Chk1 promotes the interaction between Cdh1 and βTRCP1. Immunoblot analysis of immunoprecipitates and whole-cell lysates derived from 293T cells transfected with Flag-βTRCP1, HA-Cdh1, and Myc-Chk1^L449R^ (where indicated). 30 hr post-transfection, cells were treated with the proteasome inhibitor MG132 (10 µM) for 5 hr and then harvested to do immunoprecipitation to detect the interaction between HA-Cdh1 and Flag-βTRCP1 proteins. The intensities of bound HA-Cdh1 bands were normalized to Flag-βTRCP1 , then further normalized to vector control.

**(B)** Analysis of the impact of Chk1 inhibition on Cdh1 phosphorylation in HeLa extracts. HeLa cells were synchronized and harvested in G1/S boundary, after a 2 mM hydroxyurea (HU) treatment for 16 hr. GST-Cdh1 was then incubated in extract with Chk1 inhibitor,CHIR-124 (500 nM) (where indicated) and captured on Glutathione beads. Protein bands were analyzed through Mass Spectrometry and the ratio of phosphorylated/non-phosphorylated peptides for each site was determined and normalized to the ratio in the absence of Chk1i, n=1.

(**C**) 293T cells were transfected with the indicated HA-Cdh1 constructs together with Flag-βTRCP1 and Myc-Chk1^L449R^ (where indicated). Cells were treated with 50 µg/ml cycloheximide (CHX). At the indicated time points, whole-cell lysates were prepared for immunoblot analysis. The intensities of Cdh1 bands were normalized to actin, then normalized to the t=0 time point.

**Figure S4: Chk1-Mediated Phosphorylation of Cdh1 Destabilizes the Cdh1-APC/C Interaction.**

*In-vitro* binding of wild-type or mutant Cdh1 proteins to the APC/C in G1/S extracts from HU arrested HeLa cells. Immunoblots represents both immunoprecipitated different HA-Cdh1 constructs with the relative inputs.

**Figure S5: Chk1-Mediated Phosphorylation of Cdh1 Ensures Proper Cell-Cycle Progression and Prevents DNA Damage, related to Fig. 5.**

**(A)** Representative images of nuclear size, Histone H2B-GFP, related to Figure 5A and 5B. Scale bar = 50 µm

**(B)** Representative images of EdU positive cells (top panel), total transfected (green) cells (middle panel) and merged (bottom panel) are shown related to figure 5C. Scale bar = 50 µm

**(C)** Immunofluorescence analysis of DNA damage (γH2AX). Representative images of γH2AX focus formation in U2OS cells transfected with Histone H2B-GFP and the indicated Cdh1 constructs related to Fig. 5D. Scale bar = 20 µm

**Figure S6: Chk1-Mediated Phosphorylation of Cdh1 Prevents DNA Damage, related to Fig. 5**

**(A)** Mutation of the Chk1 phosphorylation sites in Cdh1 induces DNA damage. The number of 53BP1 foci in cells. Hela cells as in (Fig 5A) were analyzed for 53BP1 foci. The graph shows the number of 53BP1 foci per GFP-positive nucleus, n>500; *p< 0.05, , ****p<0.0001 were calculated with 1-way Anova with Dunnet’s post-test.

**(B)** Immunofluorescence analysis of DNA damage (53BP1). Representative images of 53BP1 focus formation in Hela cells transfected with Histone H2B-GFP and the indicated Cdh1 constructs. Scale bar = 20 µm

**Figure S7: Model for the Role of Chk1-Mediated Cdh1 Phosphorylation on Replication Stress Response and S-Phase Entry.**

In early G1, APC/C^Cdh1^ maintains low levels of its substrates, including components required for Chk1 activitation. As cells near the G1/S transition APC/C^Cdh1^ activity diminishes (see text) and substrates accumulate, which allows activation of Chk1. Chk1 cooperates with Cyclin A and Plk1 to phosphorylate Cdh1 to create a phosphodegron which acts as a binding site for SCF^βTRCP^ leading to the ubiquitination and degradation of Cdh1. As cells leave S-phase, Plk1 targets Claspin for degradation to promote loss of Chk1 activity (see text). With decreasing Chk1 activity, Cdh1 stability increases and further inhibits additional activation of Chk1 by targeting Rad17 for degradation, thus promoting its own accumulation in G2.
