# Supplementary figures and images for: Chk1-mediated phosphorylation of Cdh1 promotes the SCFβTRCP-dependent degradation of Cdh1 during S-phase and efficient cell-cycle progression
Source: Cell Death Dis. 2020 Apr 28;11(4):298. doi: 10.1038/s41419-020-2493-1 (PMC7188793; doi:10.1038/s41419-020-2493-1)

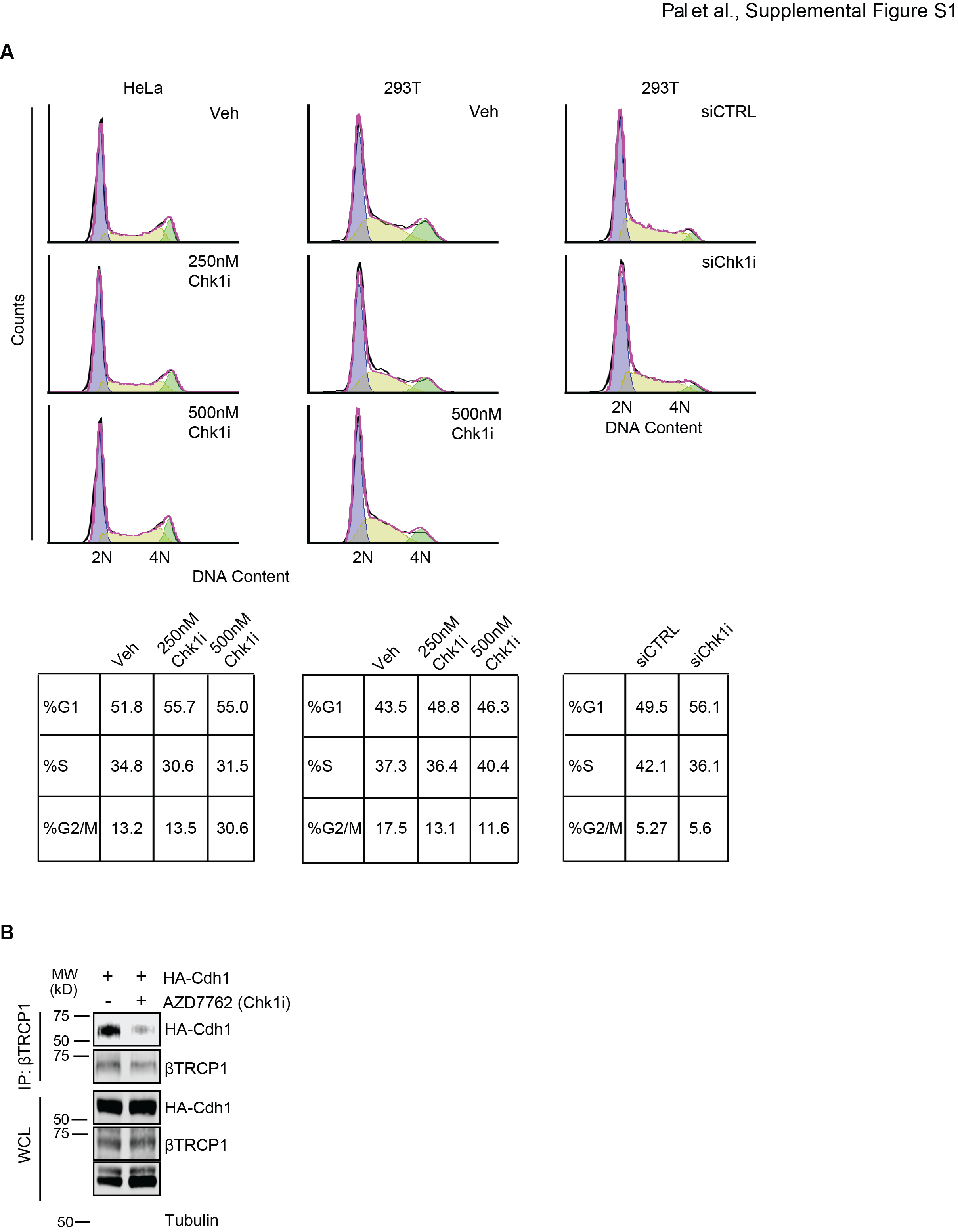

Supplement: Supplementary file 2 — Figure S1 [file 41419_2020_2493_MOESM2_ESM.png]

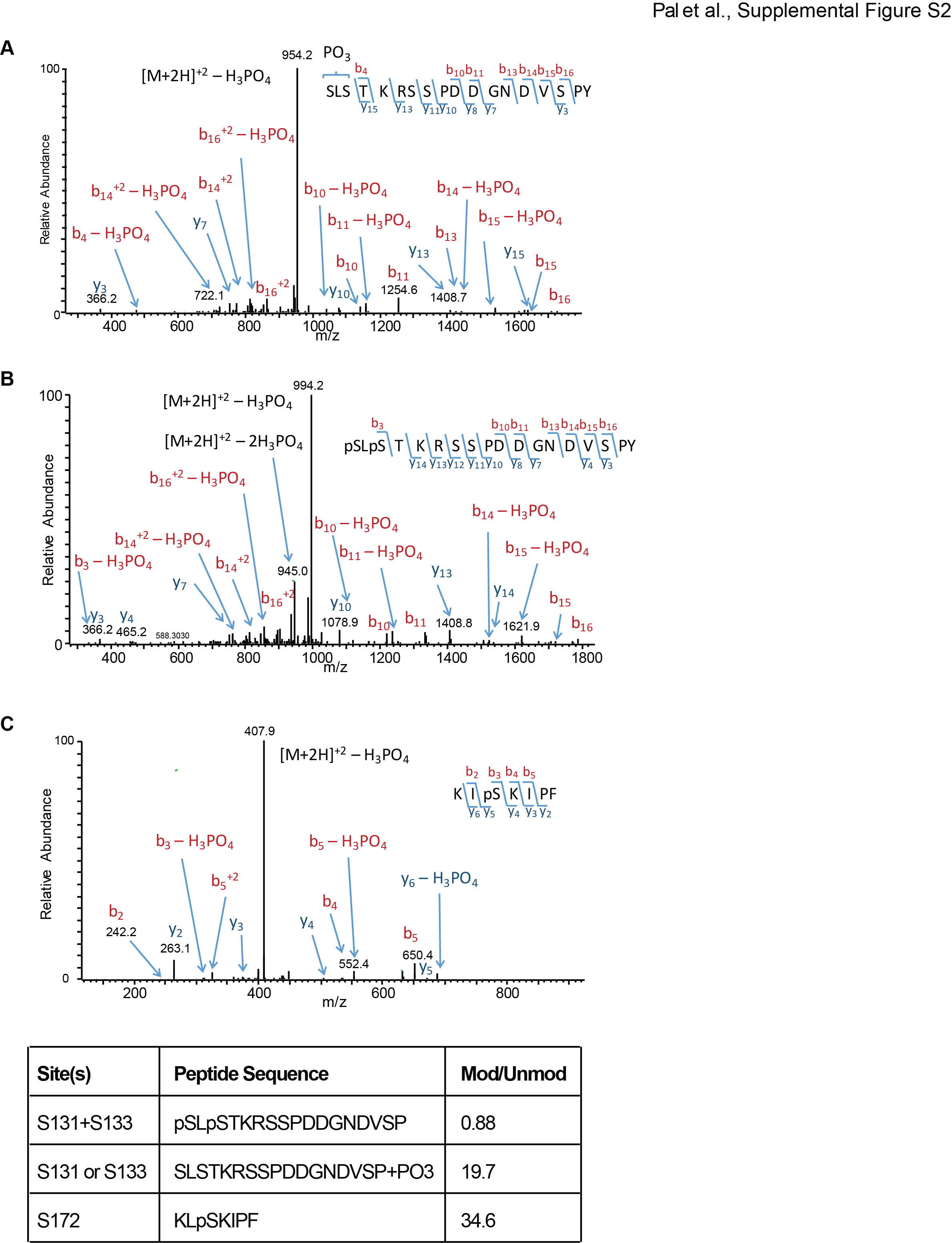

Supplement: Supplementary file 3 — Figure S2 [file 41419_2020_2493_MOESM3_ESM.png]

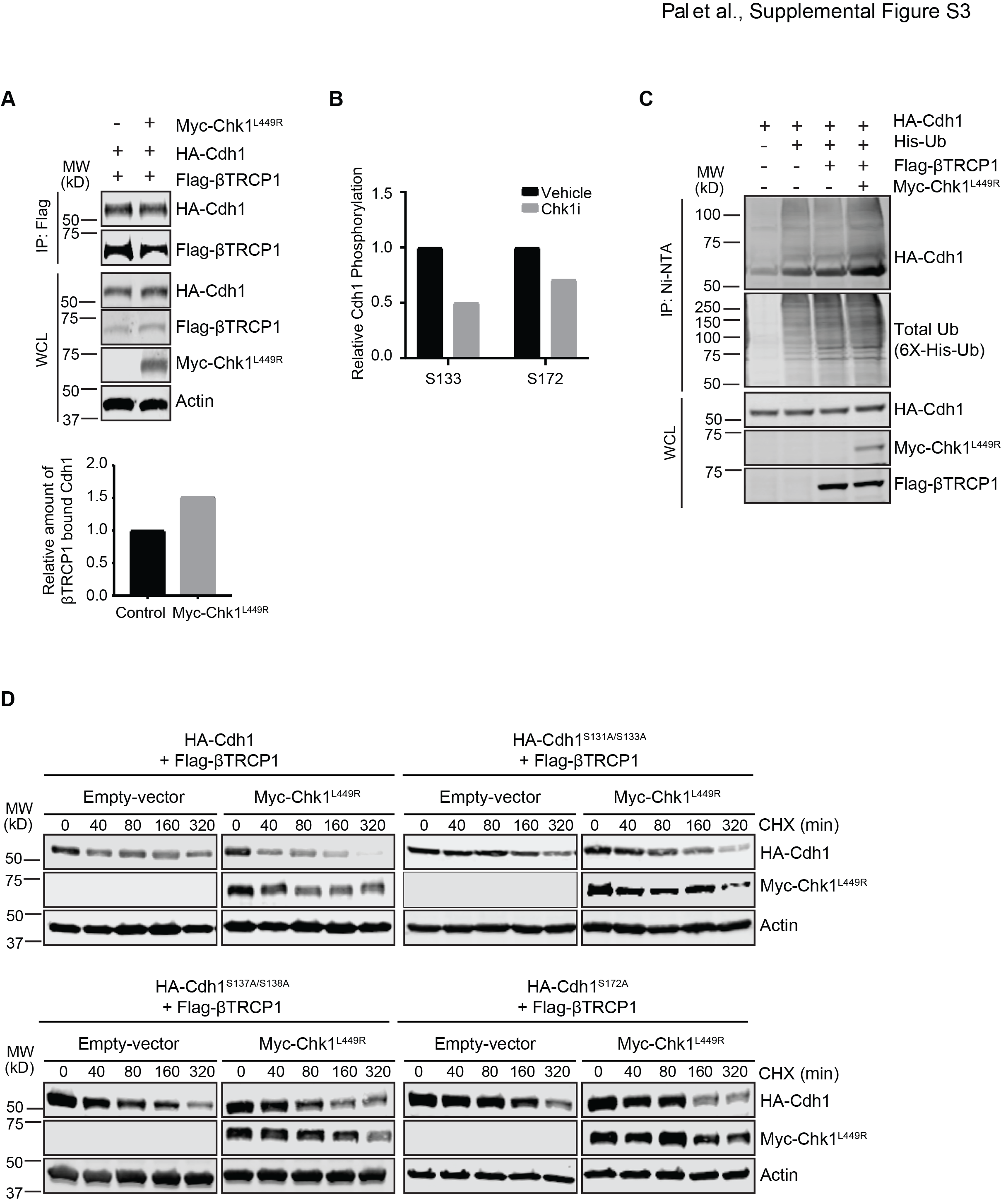

Supplement: Supplementary file 4 — Figure S3 [file 41419_2020_2493_MOESM4_ESM.png]

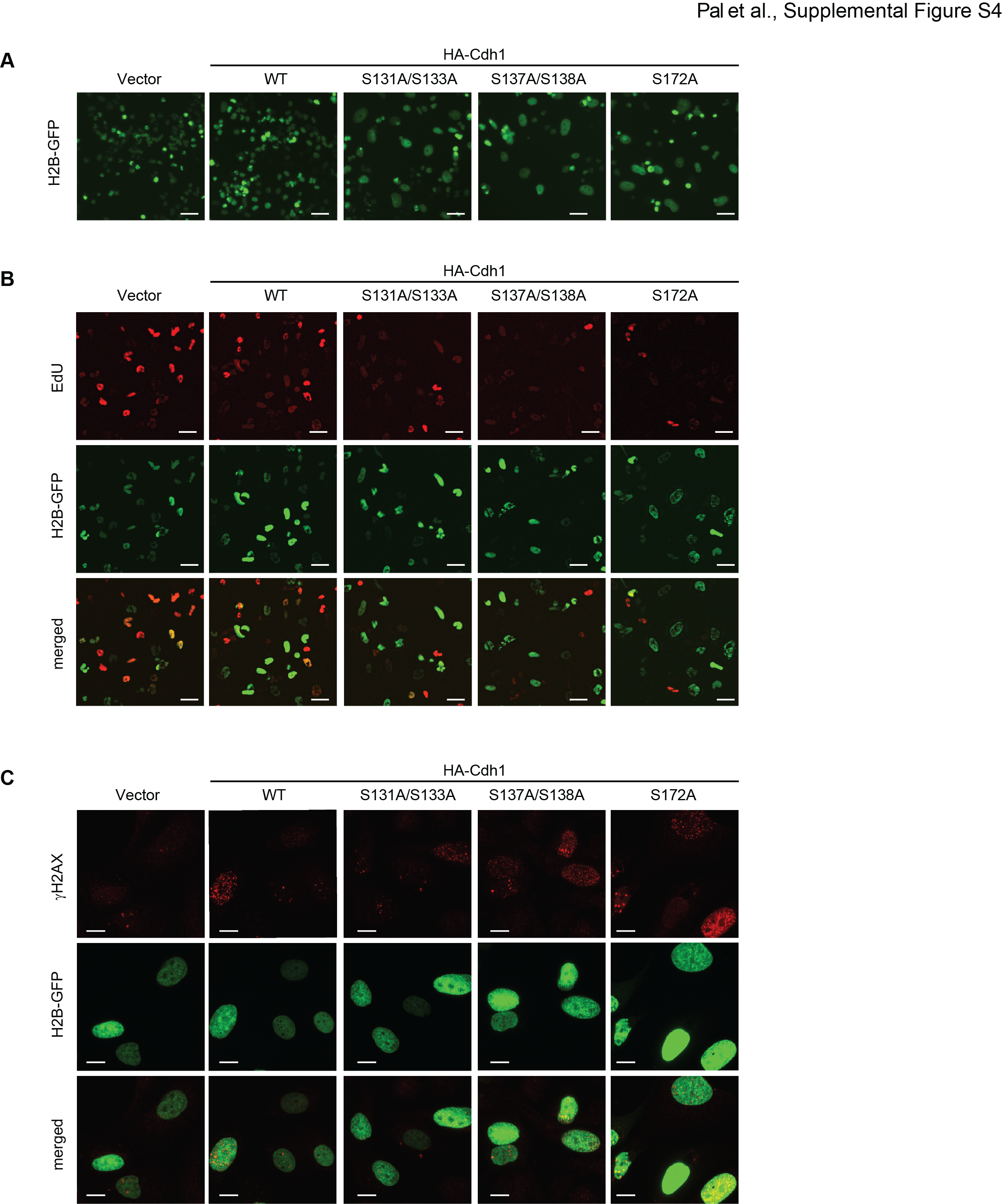

Supplement: Supplementary file 5 — Figure S4 [file 41419_2020_2493_MOESM5_ESM.png]

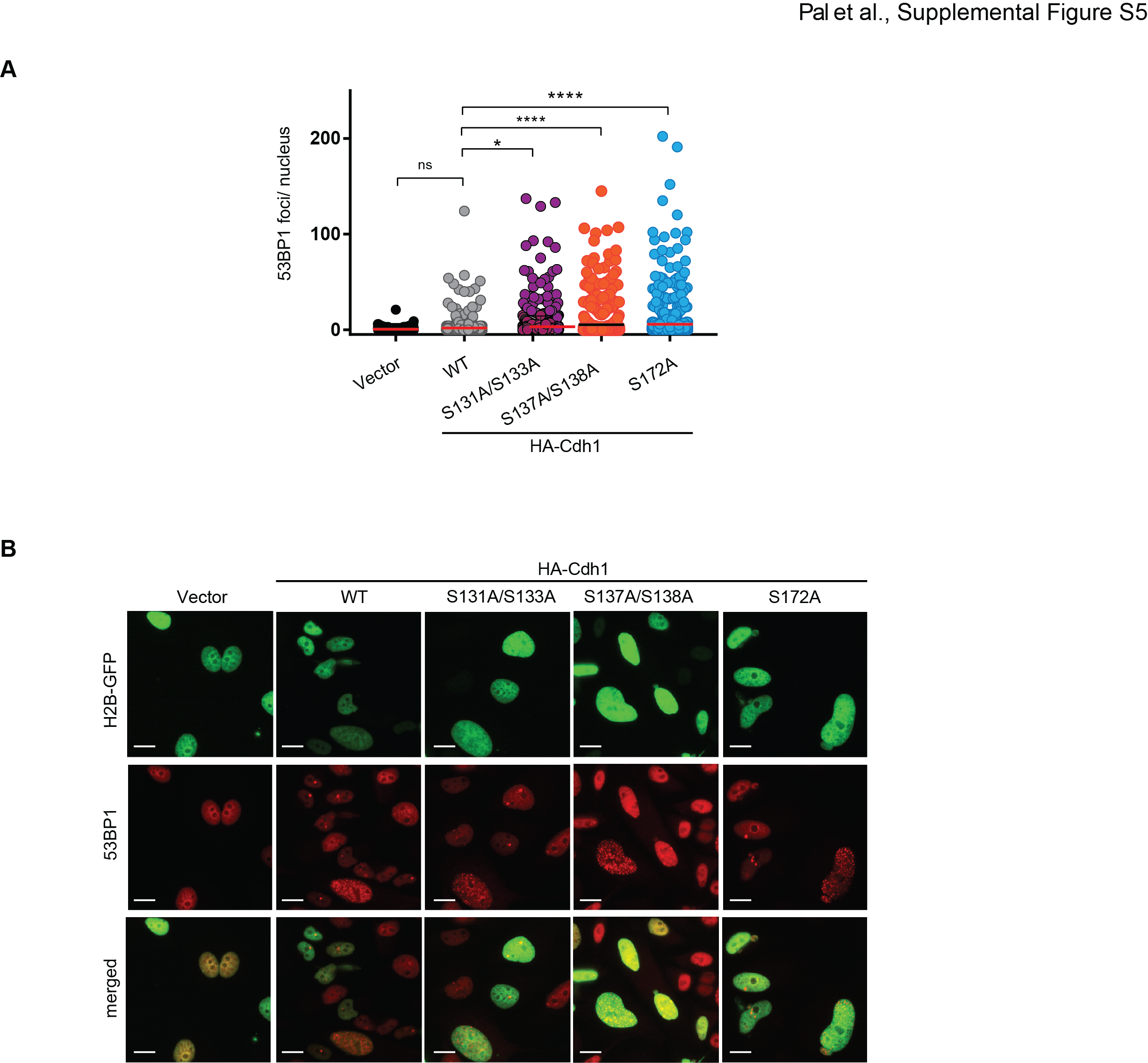

Supplement: Supplementary file 6 — Figure S5 [file 41419_2020_2493_MOESM6_ESM.png]
